# Utilization of Mycophenolic Acid, Azathioprine, Tacrolimus, Cyclosporin, Sirolimus, and Everolimus: Multinational Study

**DOI:** 10.3389/fpubh.2021.671316

**Published:** 2021-03-31

**Authors:** Majda Sahman, Snezana Mugosa, Nemanja Rancic

**Affiliations:** ^1^Department for Pharmacology, Faculty of Medicine, University of Montenegro, Podgorica, Montenegro; ^2^Institute for Medicines and Medical Devices of Montenegro, Podgorica, Montenegro; ^3^Center for Clinical Pharmacology, Military Medical Academy, Belgrade, Serbia; ^4^Faculty of Medicine of the Military Medical Academy, University of Defence, Belgrade, Serbia

**Keywords:** immunosuppressive agents, organ transplantation, pharmacoepidemiology, pharmacoeconomics, tacrolimus

## Abstract

**Background:** Organ transplantations are difficult, complicated and very expensive interventions. In order to preserve the transplanted organs, it is necessary to provide medical care to the patients in terms of immunosuppression. According to the guidelines, the first-line therapy choices for achieving immunosuppression after transplantation are tacrolimus, cyclosporine, mycophenolic acid, azathioprine, sirolimus, everolimus„ and corticosteroids. The aim of our study was to examine the utilization of this drugs in Montenegro and to compare the results with the ones from Finland, Croatia, and Serbia.

**Methods:** In our investigation we used Anatomical Therapeutic Chemical/Defined Daily Dose (ATC/DDD) methodology. Prices per DDD of drugs are presented in euros (€).

**Results:** In all observed countries, there is a positive trend in the consumption of all 6 drugs during the analyzed period. The prices per DDD of these drugs generally show a negative trend. Tacrolimus and mycophenolic acid in Montenegro recorded the largest reduction in the price per DDD. Price per one DDD of tacrolimus decreased from €13.28 in 2009 to €5.11 in 2019, thus by about 260%, and as regards mycophenolic acid, the price per one DDD decreased from €9.59 in 2009 to € 3.36 in 2019, thus by almost 300%.

**Conclusion:** Despite the reduction in the price per DDD, drugs that are used as immunosuppressants are showing increasing costs from year to year. Since these drugs are expensive, they participate in a significant percentage in the budget for medicines in each country.

## Introduction

After the transplantation, which saves and prolongs life, patients have to take immunosuppressant drugs. It helps the body not to reject the organ since immunosuppressant drugs weaken the immune system of these patients in order to reduce their body reaction to the foreign organ. It is important to distinguish three main types of immunosuppressive protocols: induction therapy, maintenance therapy and treatment of rejection (1). The choice of immunosuppressive treatment is based on efficacy, toxicity profile, individualized patient needs, etc. (2).

Immunosuppressive therapy is primarily responsible for transplant success, but its use is associated with some challenges. The issue of the costs of immunosuppressive drug therapy is particularly prominent as the cost of immunosuppressive therapy is an essential part of the overall cost of organ transplantation. For example, in the United States, the average billed charges per transplant range from $414,800 for a single kidney transplant to over $2.564 million dollars for combined heart and lung transplants (3). It is a well-known fact that immunosuppressive drugs are used throughout life, and the costs of this therapy are a significant concern not only for health insurance funds but especially for those who do not have long-term insurance coverage (4, 5). Therefore, coverage and associated funding of immunosuppressive drugs before, during, and after organ transplantation are one of the most important aspects in economic decisions in health care.

In the last few decades, advances in maintenance therapy have led to a reduction in the rate of acute organ rejection and significant progress in short-term patient survival (6). On the other hand, this trend has not been observed in long-term survival, and it has not been easy to draw conclusions due to the fact that contradictory data were reported (7, 8). Immunosuppression after solid organ transplantation represents a careful balancing act between toxicity and acute rejection (9). The goal of immunosuppressive maintenance therapy is to improve long-term outcomes while reducing the possibility of acute rejection and infection, as well as drug toxicity (10).

The literature suggests that recommended therapeutic protocols include antiproliferative agents (mycophenolic acid or azathioprine), calcineurin inhibitors (tacrolimus or cyclosporin), and the mammalian target of rapamycin (mTOR) inhibitors (sirolimus or everolimus), used often with corticosteroids and combination with antithymocyte globulin (ATG), basiliksimab, as induction therapy (4, 10–15).

Therefore, the aim of our study was to examine the utilization of mycophenolic acid, azathioprine, tacrolimus, cyclosporin, sirolimus, and everolimus in Montenegrin transplant recipients, and to compare and contrast Montenegrin data with the publically subsidized utilization data from Serbian, Croatian and Finnish.

## Materials and Methods

Retrospective cross-sectional study was performed during a 10-year period (from 2009 to 2019). The World Health Organization (WHO) Anatomical Therapeutic Chemical/Defined Daily Dose (ATC/DDD) methodology was used for the presentation and comparison of mycophenolic acid, azathioprine, tacrolimus, cyclosporin, sirolimus, and everolimus consumption statistics (16). The principle of the ATC classification system is that each unprotected name of a medicinal product (or combination of medicinal substances) corresponds to a code of seven alphanumeric characters classified into 5 levels of classification. The DDD is a technical, statistical unit of measure of drug use, which value represents the assumed average maintenance daily dose for the main indication of drug use in adults, regardless of price, pharmaceutical form, strength or size of drug packaging, and does not express recommended or actual dose of the drug used. The number of DDD per 1,000 inhabitants per day (DDD/TID) provides an insight into the number of inhabitants that used the observed drug and that were exposed to its effects during one day.

Drug consumption in Montenegro was compared with the consumption of the same drugs in Finland, Croatia and Serbia. Finland was chosen as a country with a developed pharmacotherapy practice. Montenegro, together with Croatia and Serbia, has been a part of a common state for more than half a century. Today, these three countries border each other, with Croatia being a member of the European Union (EU), and Serbia, like Montenegro, is a candidate for EU membership.

Data on the utilization and expenditure of mycophenolic acid, azathioprine, tacrolimus, cyclosporin, sirolimus, and everolimus in Montenegro were generated by the Institute for budgets and Medical Devices of Montenegro and national Health insurance fund (17). Data for the Republic of Serbia for the same time period were taken from the annual reports of the Agency for Drugs and Medical Devices of the Republic of Serbia (18, 19). Same information for the Republic of Croatia were obtained from Agency for Medicinal Products and Medical Devices of Croatia and for the Republic of Finlnad from Finnish Medicines Agency (20, 21). The given data represent the overall out-patient and in-patient use of drugs.

Microsoft Office Excel 2007 was used for statistical analysis. Trend analysis was used for data processing concerning observed period from 2009 to 2019.

## Results

Consumption of all drugs during the 11 years of the observed period is shown in Table 1, while the prices of the same drugs per DDD are shown in Table 2.

**Table 1:** Overview of the consumption of tacrolimus, azathioprine, cyclosporin, mycophenolic acid, sirolimus, and everolimus in Montenegro, Finland, Croatia, and Serbia in the period 2009–2019, presented as the number DDD/TID and euros (€).

		**2009**	**2010**	**2011**	**2012**	**2013**	**2014**	**2015**	**2016**	**2017**	**2018**	**2019**
		**DDD/TID (**€^**^)	**DDD/TID (**€^**^)	**DDD/TID (**€^**^)	**DDD/TID (**€^**^)	**DDD/TID (**€^**^)	**DDD/TID (**€^**^)	**DDD/TID (**€^**^)	**DDD/TID (**€^**^)	**DDD/TID (**€^**^)	**DDD/TID (**€^**^)	**DDD/TID (**€^**^)
Tacrolimus	MNE	0.05 (150.25)	0.05 (144.83)	0.05 (119.60)	0.05 (64.40)	0.06 (130.80)	0.06 (155.86)	0.07 (187.47)	0.09 (187.47)	0.08 (159.77)	0.10 (127.72)	0.11 (127.30)
	FIN	0.12 (2,905.00)	0.13 (2,984.00)	0.15 (3,427.00)	0.16 (3,751.00)	0.14 (2,907.00)	0.17 (3,524.00)	0.22 (4,539.00)	0.24 (4,879.00)	0.28 (5,639.00)	0.34 (6,721.00)	0.39 (7,378.00)
	CRO	0.04 (737.15)	0.08 (1,334.99)	0.09 (1,523.85)	0.12 (1,974.56)	0.19 (2,160.22)	0.15 (1,945.35)	0.18 (2,453.27)	0.20 (2,577.19)	0.21 (2,498.61)	0.24 (2,824.21)	0.28 (3,341.05)
	SRB	0.04 (1,170.87)	0.04 (1,222.42)	0.05 (1,412.10)	0.05 (1,273.88)	0.05 (1,187.68)	0.06 (1,198.76)	0.07 (1,474.81)	0.07 (1,488.72)	0.08 (1,775.78)	0.10 (1,900.15)	0.09 (1,679.32)
Azathioprine	MNE	0.19 (15.09)	0.19 (13.26)	0.18 (18.71)	0.25 (33.63)	0.25 (33.17)	0.29 (39.67)	0.26 (33.21)	0.31 (33.21)	0.36 (43.99)	0.36 (44.23)	0.36 (43.23)
	FIN	1.08 (752.00)	1.09 (760.00)	1.19 (851.00)	1.18 (850.00)	0.93 (675.00)	1.25 (904.00)	1.25 (909.00)	1.41 (1,029.00)	1.39 (997.00)	1.48 (1,078.00)	1.17 (861.00)
	CRO	0.21 (248.04)	0.24 (218.35)	0.29 (242.56)	0.31 (260.82)	0.35 (291.37)	0.36 (295.04)	0.39 (322.82)	0.40 (329.99)	0.41 (333.18)	0.40 (311.12)	0.43 (338.84)
	SRB	0.15 (246.00)	–	–	0.03 (330.64)	0.03 (455.40)	0.03 (390.16)	0.04 (517.92)	0.03 (389.53)	0.41 (552.32)	0.41 (545.47)	0.48 (634.77)
Cyclosporin	MNE	0.07 (138.81)	0.07 (68.96)	0.08 (97.94)	0.07 (77.09)	0.08 (99.32)	0.07 (82.88)	0.06 (80.41)	0.07 (80.41)	0.06 (71.94)	0.06 (72.56)	0.06 (68.16)
	FIN	0.45 (8,390.00)	0.45 (8,293.00)	0.44 (8,159.00)	0.43 (8,253.00)	0.44 (8,020.00)	0.44 (7,678.00)	0.44 (7,672.00)	0.42 (7,051.00)	0.40 (6,055.00)	0.40 (4,933.00)	0.39 (4,744.00)
	CRO	0.12 (1,326.35)	0.16 (1,603.97)	0.18 (1,764.67)	0.17 (1,727.33)	0.18 (1,570.71)	0.16 (1,524.78)	0.18 (1,625.16)	0.18 (1,560.77)	0.17 (1,341.99)	0.16 (1,307.51)	0.16 (1,276.46)
	SRB	0.05 (970.64)	0.04 (827.04)	0.05 (883.56)	0.06 (861.49)	0.05 (908.16)	0.04 (793.86)	0.05 (676.04)	0.06 (813.58)	0.05 (778.28)	0.05 (737.47)	0.05 (751.61)
Mycophenolic acid	MNE	0.10 (217.06)	0.10 (213.90)	0.10 (156.56)	0.10 (144.99)	0.13 (183.71)	0.13 (177.50)	0.14 (193.50)	0.15 (193.50)	0.15 (161.99)	0.15 (124.87)	0.18 (136.99)
	FIN	0.29 (5,420.00)	0.32 (5,884.00)	0.32 (5,311.00)	0.35 (5,338.00)	0.29 (4,502.00)	0.34 (5,218.00)	0.39 (6,031.00)	0.40 (5,152.00)	0.43 (4,419.00)	0.44 (4,577.00)	0.47 (4,743.00)
	CRO	0.20 (3,144.69)	0.26 (3,645.52)	0.30 (4,082.20)	0.77 (3,636.26)	1.43 (5,305.57)	0.43 (5,318.55)	0.42 (4,022.40)	0.47 (4,592.25)	0.44 (4,325.49)	0.48 (4,611.18)	0.50 (4,722.74)
	SRB	0.07 (1,793.76)	0.07 (1,744.48)	0.08 (1,718.41)	0.01 (1,115.69)	0.02 (1,298.67)	0.02 (1,338.40)	0.05 (1,016.12)	0.03 (977.74)	0.05 (992.90)	0.08 (1,131.53)	0.08 (1,127.02)
Sirolimus	MNE	0.00* (11.08)	0.00* (8.42)	0.00* (3.89)	0.00* (2.31)	–	0.00* (6.99)	0.00* (3.60)	0.00* (3.60)	0.00* (7.99)	0.00* (6.19)	0.00* (7.80)
	FIN	0.00* (77.00)	0.00* (86.00)	0.00* (88.00)	0.00* (86.00)	0.00* (81.00)	0.00* (91.00)	0.01 (113.00)	0.01 (124.00)	0.01 (149.00)	0.01 (159.00)	0.01 (191.00)
	CRO	0.00* (87.51)	0.00* (82.42)	0.00* (92.03)	0.00* (88.65)	0.01 (86.45)	0.01 (95.31)	0.01 (87.68)	0.01 (93.80)	0.01 (90.43)	0.01 (84.79)	0.00* (74.33)
	SRB	0.01 (214.55)	0.01 (194.75)	0.00* (124.27)	0.00* (94.75)	0.00* (113.95)	0.00* (101.26)	–	0.00* (59.54)	0.00* (72.22)	0.00* (85.21)	0.00* (78.90)
Everolimus	MNE	–	–	0.00* (12.52)	0.00* (66.59)	0.00* (122.63)	0.00* (179.08)	0.00* (113.55)	0.00* (113.55)	0.00* (144.30)	0.00* (152.76)	0.00* (328.61)
	FIN	0.00* (29.00)	0.00* (411.00)	0.00* (1,191.00)	0.00* (1,772.00)	0.00* (2,083.00)	0.00* (2,180.00)	0.00* (2,280.00)	0.00* (1,976.00)	0.00* (1,865.00)	0.00* (2,065.00)	0.00* (1,711.00)
	CRO	0.00* (32.79)	0.00* (24.73)	0.00* (20.22)	0.01 (287.56)	0.01 (652.68)	0.02 (1,281.74)	0.03 (1,589.99)	0.04 (2,143.63)	0.05 (2,118.39)	0.06 (1,914.11)	0.07 (1,537.37)
	SRB	0.00* (0.55)	0.00* (3.02)	0.00* (3.84)	0.00* (24.25)	0.00* (44.40)	0.01 (203.74)	0.01 (243.50)	0.01 (279,87)	0.01 (752.07)	0.01 (959.97)	0.01 (1,211.05)

0.00^*^, consumption below 0.01%.

** *Cost expressed in euros as actual cost/1000*.

*MNE, Montenegro; FIN, Finland; CRO, Croatia; SRB, Serbia*.

**Table 2:** Overview of the prices of 1 DDD tacrolimus, azathioprine, cyclosporin and mycophenolic acid in Montenegro, Finland, Croatia and Serbia in the period 2009–2019, expressed in euros (€).

		**2009**	**2010**	**2011**	**2012**	**2013**	**2014**	**2015**	**2016**	**2017**	**2018**	**2019**
		€	€	€	€	€	€	€	€	€	€	€
Tacrolimus	MNE	13.28	12.80	10.57	5.69	9.63	11.48	11.83	9.20	8.83	5.64	5.11
	FIN	12.28	11.65	11.59	11.89	10.53	10.52	10.47	10.31	10.22	10.03	9.60
	CRO	12.62	11.43	11.60	11.27	7.79	8.88	9.34	8.83	8.15	8.06	8.17
	SRB	11.30	11.79	10.90	9.83	9.17	7.71	8.13	8.21	8.57	7.33	7.20
Azathioprine	MNE	0.35	0.31	0.46	0.59	0.59	0.60	0.56	0.47	0.54	0.54	0.53
	FIN	0.35	0.35	0.36	0.37	0.37	0.37	0.37	0.37	0.36	0.37	0.37
	CRO	0.81	0.62	0.57	0.58	0.57	0.56	0.57	0.57	0.56	0.53	0.54
	SRB	0.63	−	−	4.25	5.86	5.02	5.00	5.01	0.52	0.51	0.51
Cyclosporin	MNE	8.76	4.35	5.41	4.87	5.49	5.23	5.92	5.08	5.30	5.34	5.02
	FIN	9.46	9.35	9.41	9.74	9.25	8.85	8.85	8.52	7.68	6.26	6.17
	CRO	7.57	6.87	6.71	6.96	5.98	6.53	6.18	5.94	5.41	5.60	5.46
	SRB	7.49	7.98	6.82	5.54	7.01	7.66	5.22	5.23	6.01	5.69	5.80
Mycophenolic acid	MNE	9.59	9.45	6.92	6.41	6.24	6.03	6.11	5.70	4.77	3.68	3.36
	FIN	9.48	9.33	8.42	7.97	7.88	7.79	7.85	6.53	5.21	5.28	5.12
	CRO	10.77	9.60	9.32	3.23	2.71	8.47	6.56	6.69	6.73	6.58	6.47
	SRB	9.89	9.62	0.83	43.05	25.06	25.82	7.84	12.58	7.66	5.46	5.44

*MNE, Montenegro; FIN, Finland; CRO, Croatia; SRB, Serbia*.

The consumption of tacrolimus in all observed countries is increasing. In 2019, the consumption of tacrolimus in Montenegro is the same as in Finland at the beginning of the observed period (2009). The highest costs for tacrolimus were recorded in Finland. In Montenegro, the costs for tacrolimus are quite low. The price per one DDD in Montenegro in 2009 was around €13, and in 2019 it was around €5, thus we find almost three times reduction in price per one DDD. We notice a large difference in the price per one DDD of tacrolimus in the observed countries.

As with tacrolimus, the highest consumption of azathioprine was recorded in Finland. At the same time, the largest funds for azathioprine are spent in Finland. During the last few years, the price per one DDD of azathioprine in Croatia and Serbia was quite similar to the price per one DDD in Montenegro.

The highest consumption of cyclosporine during the observed period was recorded in Finland, with a slight downward trend. The largest funds for this drug were allocated in Finland. The consumption of this drug in Montenegro, similarly as in Serbia, was quite small and ranged around 0.06 DDD/TID.

During the last 8 years of the observed period, mycophenolic acid was mostly used in Croatia. Very similar consumption of this drug can also be seen in Finland. In Montenegro, the consumption of mycophenolic acid shows a positive trend. During the last 6 years of the observed period, the price per one DDD of mycophenolic acid was the lowest in Montenegro (Table 2).

The consumption of everolimus and sirolimus in all 4 countries was extremely low. However, the data on the funds spent indicate that these are the drugs that significantly affect the budget for medicines in all 4 countries.

## Discussion

Organ transplantations are difficult, complicated, and very expensive interventions. In order to preserve the transplanted organs, it is necessary to provide medical care to the transplanted patients in terms of immunosuppression. According to the guidelines, the first-line therapy choices for achieving immunosuppression after kidney transplantation include tacrolimus or cyclosporine, mycophenolic acid and corticosteroids (22, 23). In some patients, azathioprine can still be used as one of the oldest immunosuppressive drugs, as well as sirolimus and everolimus, two relatively new but also expensive drugs. The therapy that patients receive after transplantation is expensive. If we take into account the lifelong use of these drugs, then we come to the conclusion that after an expensive intervention, there are still high costs of maintaining immunosuppression. After transplantation, immunosuppressive drugs are issued to patients in Montenegro at the expense of the health insurance fund. Long-term expensive therapy has a significant impact on the budget for medicines in this country. In 2016, around €68,000,000 was spent on medicines and medical devices (approximately €50,000,000 only on medicines), while in 2017 and 2018 about €70,000,000 (24). If we look at the consumption of these drugs, we can see that in 2016, 1.22% of the budget for medicines in Montenegro was allocated for these 6 preparations and in 2018, 0.81% of the budget. If the same funds for medicines were spent in 2019 as in 2018, the share of these 6 products in the total budget for medicines in Montenegro would be 1.10%. As it can be noticed, the listed drugs consume significant budget funds. It is encouraging that the utilization of some of these 6 drugs recorded a steady increase, while at the same time the amount of funds spent decreased. Their prices per DDD in Montenegro have dropped.

Tacrolimus has been the basis of immunosuppressive therapeutic regimens for more than two decades. According to SmPC, it is used in the prophylaxis of allogeneic kidney or liver transplant rejection in adult patients and in the treatment of allogeneic transplant rejection when there is a resistance to other immunosuppressants in adult patients (25). Although effective, it is a drug with a narrow therapeutic index and serious adverse drug reactions. It showed nephrotoxic and neurotoxic effects (26). Consumption of this drug in the observed period recorded a constant increase in all observed countries. In Montenegro, the utilization of this drug doubled from 2009 to 2019. However, compared to Finland, we found that the consumption of this drug in Montenegro was 3–4 times lower in the last few years.

There are data on the possible safe therapeutic switch from reference form to the generic form of tacrolimus in patients with a kidney transplant (27). The increase in consumption in Montenegro could be attributed to the appearance of generic forms of this drug, which is justified. The total costs for tacrolimus increased at the beginning of the observed period together with the increase of consumption. However, since 2015, total costs have decreased, regardless of consumption increase. In other words, the price per one DDD of tacrolimus decreased significantly during the observed period. Compared to 2009, it decreased by about 260% in 2019 (from €13.28 to €5.11). We do not find such a large price reduction in other countries. The cost of monthly therapy with tacrolimus in 2019 in Montenegro was €153.41, in Finland €287.94, in Croatia €245.19 and in Serbia €216.00. We can assume that the reason for such a large reduction in the price per one DDD is also the emergence of a larger number of generic drugs in Montenegro.

Cyclosporine had a lower price per DDD compared to tacrolimus during the observed period. According to the guidelines, one of these two drugs is used after the organ transplantation. However, studies showed that the number of patients who did not reject the transplant acutely was higher with tacrolimus than with cyclosporine. We certainly cannot ignore the fact that tacrolimus can cause impaired renal function and induce insulin-dependent diabetes mellitus (28). The consumption of cyclosporine was fairly uniform during the observed period and was similar to the consumption of tacrolimus in Montenegro. However, since 2015, cyclosporine consumption has been discretely lower than tacrolimus consumption. Cyclosporine is a drug that is used in many other indications in addition to transplantation (29). Considering the fact that acute transplant rejection is less common with tacrolimus compared to cyclosporine, the more frequent use of tacrolimus is then justified, even though it has a slightly higher price per DDD. According to the prices from 2019, the cost of monthly therapy with tacrolimus in Montenegro amounted to €153.41, while the cost of monthly therapy with cyclosporine amounted to €150.60.

Mycophenolic acid is an immunosuppressant that has been an integral part of the protocol for achieving immunosuppression in post-transplant patients since 1995. Initial research showed that this drug has reduced the frequency of acute transplant rejection by as much as 50% (23). This drug recorded a slight increase in consumption in Montenegro from 2009 to 2019. In the last few years, similarly to tacrolimus, the increase in consumption of this drug has been accompanied by a reduction in its price. The price per one DDD decreased from €9.59 in 2009 to €3.36 in 2019, thus by almost 300%. We do not find such reduction in price per one DDD of mycophenolic acid in any other country. Monthly mycophenolic acid therapy cost decreased from €287.75 to €100.89. The cost of monthly therapy with mycophenolic acid in Finland is €153.60, in Croatia €194.09 and in Serbia €163.08.

Sirolimus is a drug that was introduced into therapy in patients with impaired renal coil function, with the finding of advanced fibrosis confirmed on the basis of the biopsy material or by echosonography, with the aim of slowing the progression of chronic allograft nephropathy (30). Due to the high price per one DDD (around €10), this drug is not the first-choice immunosuppressant after transplantation (31). The cost of monthly therapy with this drug in Montenegro would be around €300. Therefore, its consumption is extremely low in Montenegro and is below 0.01 DDD/TID. The results of a study conducted on kidney transplant patients showed that there was no difference in the five-year graft survival in tacrolimus-treated patients compared to sirolimus-treated patients (32).

According to SmPC, Everolimus is used in kidney cancer, advanced breast cancer, neuroendocrine tumor of the pancreas, and gastrointestinal and lung tumors (33). However, numerous studies showed positive outcomes of switching from tacrolimus or cyclosporine to everolimus in transplanted patients (34). In Serbia, everolimus is on the List of Drugs (issued at the expense of the Fund) for the prevention of organ rejection in adult patients with low or moderate immune risk who received an allogeneic kidney or heart transplant (Z94.0; Z94.1) (35). In Montenegro, this drug records an extremely low consumption (below 0.01 DDD / TID). On the other hand, the funds spent on this drug in 2015 amounted to €113,552.69, and by 2019 they increased by about 290%; more precisely, in 2019, a total of €328,608.00 was spent on this drug. The price per one DDD of sirolimus in Montenegro is around €8.6 (31). According to many authors, the use of everolimus has no advantage over tacrolimus. A study by Bouamar et al. showed that switching from tacrolimus to everolimus-based immunosuppression with competitive withdrawal of prednisolone three months after kidney transplantation (from living donors) results in an unacceptably high risk of acute transplant rejection (36).

Azathioprine is a drug that is used alone or in combination with corticosteroids to achieve immunosuppression. It is indicated for improving the graft survival, such as the kidney, heart and liver graft survival. It also reduces the need for corticosteroids in kidney transplant recipients. This drug is also used in a number of other indications that require the achievement of immunosuppression in patients (37). The consumption of azathioprine in Montenegro in the observed period recorded an increase of almost 200%, from 0.19 in 2009 to 0.36 DDD/TID in 2019. In the same period, the costs for this drug increased about 3 times, which directly affected the increase in the price per one DDD. Thus, the price per one DDD of this drug in 2009 was €0.35, while 10 years later it was €0.53. We can notice that out of six analyzed drugs, only azathioprine shows an increase in the price of one DDD. However, despite the increase in the price per one DDD, the cost of monthly therapy with this drug still remains the most favorable. According to the obtained data, azathioprine would have the smallest impact on the budget for drugs in Montenegro; however, some of the clinical studies do not classify this drug as the first-choice immunosuppressant after transplantation. There is a significant risk of developing squamous cell carcinoma of the skin, especially when using immunosuppressants after lung transplantation. That risk is higher if azathioprine is used, compared to mycophenolate mofetil—a prodrug of mycophenolic acid. This could be a significant reason for replacing azathioprine with mycophenolic acid, regardless of the observed pharmacoeconomic benefits this drug provides (38).

In general, we can state that an increase in consumption can be noticed in almost all analyzed preparations in all 4 countries during the observed period we find. The exception is cyclosporine, where we see a discrete decline in consumption, but also a reduction in the cost of this drug in all 4 countries.

## Conclusion

Drugs that are taken as immunosuppressants are showing increasing consumption from year to year. The reason for that is, among other things, the increasing number of transplants, after which the long-term therapy with these drugs is necessary. Since these drugs are expensive, they participate in a significant percentage in the budget for medicines in each country. In Montenegro, over 1% of funds intended for medicines has been spent on 6 analyzed drugs in the last few years.

## Limitation of the Study

The limitation of this study is certainly the data on extremely low consumption of everolimus and sirolimus in most countries (below 0.01 DDD/TID). We can assume that the data we had are not completely accurate, which further made it impossible to calculate the price per one DDD of these 2 drugs.

## Data Availability Statement

The raw data supporting the conclusions of this article will be made available by the authors, without undue reservation.

## Author Contributions

NR: conceptualization. MS, SM, and NR: methodology. MS and SM: formal analysis. MS, SM, and NR: investigation. MS and SM: writing-original draft preparation. NR: writing-review and editing. All authors have read and agreed to the published version of the manuscript.

## Conflict of Interest

The authors declare that the research was conducted in the absence of any commercial or financial relationships that could be construed as a potential conflict of interest.
